# A summary index derived from Kinect to evaluate postural abnormalities severity in Parkinson’s Disease patients

**DOI:** 10.1038/s41531-022-00368-x

**Published:** 2022-08-02

**Authors:** Ronghua Hong, Tianyu Zhang, Zhuoyu Zhang, Zhuang Wu, Ao Lin, Xiaoyun Su, Yue Jin, Yichen Gao, Kangwen Peng, Lixi Li, Lizhen Pan, Hongping Zhi, Qiang Guan, Lingjing Jin

**Affiliations:** 1 Neurotoxin Research Center, Key Laboratory of Spine and Spinal Cord Injury Repair and Regeneration of Ministry of Education, Neurological Department of Tongji Hospital, School of Medicine, Tongji University, Shanghai, China; 2 IFLYTEK Suzhou Research Institute, Suzhou, China; 3 Department of Neurology and Neurological Rehabilitation, Shanghai Yangzhi Rehabilitation Hospital, School of Medicine, Tongji University, Shanghai, China; 4 Shanghai Clinical Research Center for Aging and Medicine, Shanghai, China

**Keywords:** Parkinson's disease, Neurodegeneration, Diagnostic markers

## Abstract

Postural abnormalities are common disabling motor complications affecting patients with Parkinson’s disease (PD). We proposed a summary index for postural abnormalities (IPA) based on Kinect depth camera and explored the clinical value of this indicator. Seventy individuals with PD and thirty age-matched healthy controls (HCs) were enrolled. All participants were tested using a Kinect-based system with IPA automatically obtained by algorithms. Significant correlations were detected between IPA and the Movement Disorder Society-Sponsored Revision of the Unified Parkinson’s Disease Rating Scale (MDS-UPDRS) total score (*r*_s_ = 0.369, *p* = 0.002), MDS-UPDRS-III total score (*r*_**s**_ = 0.431, *p* < 0.001), MDS-UPDRS-III 3.13 score (*r*_s_ = 0.573, *p* < 0.001), MDS-UPDRS-III-bradykinesia score (*r*_s_ = 0.311, *p* = 0.010), the 39-item Parkinson’s Disease Questionnaire (PDQ-39) (*r*_s_ = 0.272, *p* = 0.0027) and the Berg Balance Scale (BBS) score (*r*_s_ = −0.350, *p* = 0.006). The optimal cut-off value of IPA for distinguishing PD from HCs was 12.96 with a sensitivity of 97.14%, specificity of 100.00%, area under the curve (AUC) of 0.999 (0.997–1.002, *p* < 0.001), and adjusted AUC of 0.998 (0.993–1.000, *p* < 0.001). The optimal cut-off value of IPA for distinguishing between PD with and without postural abnormalities was 20.14 with a sensitivity, specificity, AUC and adjusted AUC of 77.78%, 73.53%, 0.817 (0.720–0.914, *p* < 0.001), and 0.783 (0.631–0.900, *p* < 0.001), respectively. IPA was significantly correlated to the clinical manifestations of PD patients, and could reflect the global severity of postural abnormalities in PD with important value in distinguishing PD from HCs and distinguishing PD with postural abnormalities from those without.

## Introduction

Postural abnormalities are disabling motor complications affecting patients with Parkinson’s disease (PD) and become increasingly severe as the disease progresses^1–3^. The most recognized type of postural abnormalities in PD patients is the classic stooped posture, with flexion of the hips and knees, and rounding of the shoulders, which differs them from general population^2,4^. Moreover, an important subset of patients present with more severe abnormalities of spinal alignment including sagittal abnormalities: camptocormia and anterocollis^5,6^; frontal abnormalities: Pisa syndrome and scoliosis^2,7^. Some patients even suffer from a combination of several types of postural abnormalities. Since postural abnormalities in PD subjects usually develop insidiously over months to years before they become obvious^8,9^, early recognition of them facilitates the diagnosis of the disease, as well as the prompt intervention to avoid worse outcomes.

In clinical practice, there are various methods for evaluating abnormal posture of PD, such as clinical scale, wall goniometer, and photo-based measurement^10–12^. The most commonly used clinical scale for evaluating abnormal posture of PD is the 13th item of the third part of Movement Disorder Society-Sponsored Revision of the Unified Parkinson’s Disease Rating Scale (MDS-UPDRS-III 3.13). However, the 5-class ordinal scale gives only a broad classification of postures and is not suitable for a more detailed description because of its insensitivity to small changes which may be clinically relevant^10,13^. In addition, simple geometric parameters, angles, are generally examined to evaluate postural abnormalities in PD. Conventionally, three common methods, including total camptocormia (TCC) angle, upper camptocormia (UCC) angle, and lower camptocormia (LCC) angle are used to assess the severity of camptocormia^6,12,14^. Similarly, the drop head angle (DHA) characterizes the severity of anterocollis while the Pisa angle or the lateral trunk bending (LTB) angle serves to assess PD patients with LTB like Pisa syndrome and scoliosis^4,15–17^. Though the wall goniometer method and photo-based measurement method can provide accurate and quantitative measurement of the above angles, they only reflect the severity of postural abnormalities of a certain plane and do not offer a global assessment of postural orientation quality of the patients^4,12,15,16,18,19^. The global and quantitative assessment of posture is urgently required to monitor the progress of the disease and measure treatment effects.

**Fig. 1:**
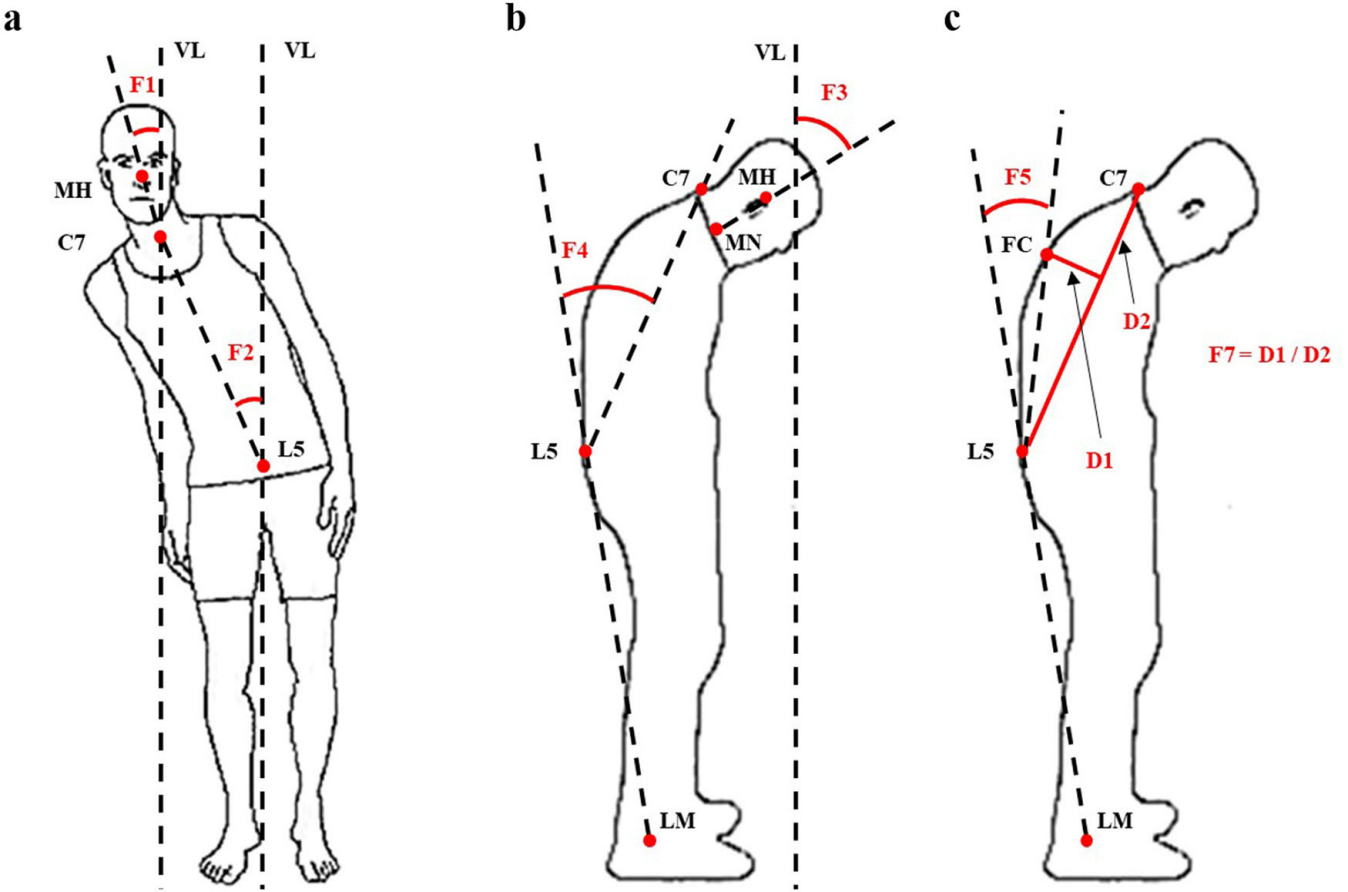
Illustration of the six selected features F1, F2, F3, F4, F5, and F7 (modified from ref. ^27^). F1: lateral flexion angle of head, the angle between the connecting line of MH and C7 on the coronal plane and VL (**a**); F2: lateral flexion angle of trunk, the angle between the connecting line of C7 and L5 on the coronal plane and VL (**a**); F3: forward flexion angle of head, the angle between the connecting line of MH and MN on the sagittal plane and VL (**b**); F4: total forward flexion angle of trunk, the angle between the connecting line of L5 and LM and the connecting line of C7 and L5 on the sagittal plane (**b**); F5: forward flexion angle of trunk at the waist, the angle between the connecting line of L5 and LM and the connecting line of FC and L5 on the sagittal plane (**c**); F7: F7 was normalized as “D1/D2” with “%” as unit, to eliminate the effect of anthropometrical difference. D1 and D2 referred to the distance between FC and the connecting line of C7 and L5, and distance between C7 and L5 on the sagittal plane, respectively (**c**). *MH* the midpoint of head, *MN* the midpoint of neck, *VL* the vertical line of the ground, *C7* the 7th cervical spinous process, *L5* the 5th lumbar spinous process, *LM* lateral malleolus, *FC* vertebral fulcrum which indicates the most convex point of the vertebra.

In the last decade, three-dimensional (3D) stereophotogrammetry including Kinect depth camera has been widely used to provide objective information about main joint motions on the three planes of movement^20–22^. To further facilitate the interpretation of these large amount of kinematic data, indices such as Gait Profile Score (GPS)^23,24^, Trunk Profile Score (TPS)^25^, and Arm Profile Score (APS)^26^ have been proposed and show promising clinical values. Inspired by these attempts, we recently developed an intelligent evaluation system to assess postural abnormalities in PD based on Kinect and machine learning^27^. The automated and accurate assessment of postural abnormalities for each PD patient was realized with only six selected features F1, F2, F3, F4, F5, and F7 (Fig. 1). Their feature importance in the constructed decision tree model was 13.2%, 12.6%, 16.5%, 11.3%, 6.7%, and 40% severally^27^. Actually, F2, F4, and F5 here shared the same definitions of LTB, TCC, and LCC angles, respectively, which reflected the severity of trunk abnormality^12,16^. F1 and F3 reflected the severity of head and neck abnormality similar to DHA^4,16^. F7 was a normalized feature which offered a general assessment in patients with both lower and upper camptocormia^27^. In this study, we proposed a summary index, the index for postural abnormalities (IPA), which was a combination of all these features (F1, F2, F3, F4, F5, and F7). We explored the correlations between IPA and other clinical manifestations of PD patients and discriminated between participants with different severity of postural abnormalities. In our hypothesis, the IPA is a useful method to assess the clinical severity of postural abnormalities in PD globally with important value in distinguishing PD from healthy general population and distinguishing between PD with and without postural abnormalities.

## Results

### The demographic and clinical characteristics of the participants

**Fig. 2:**
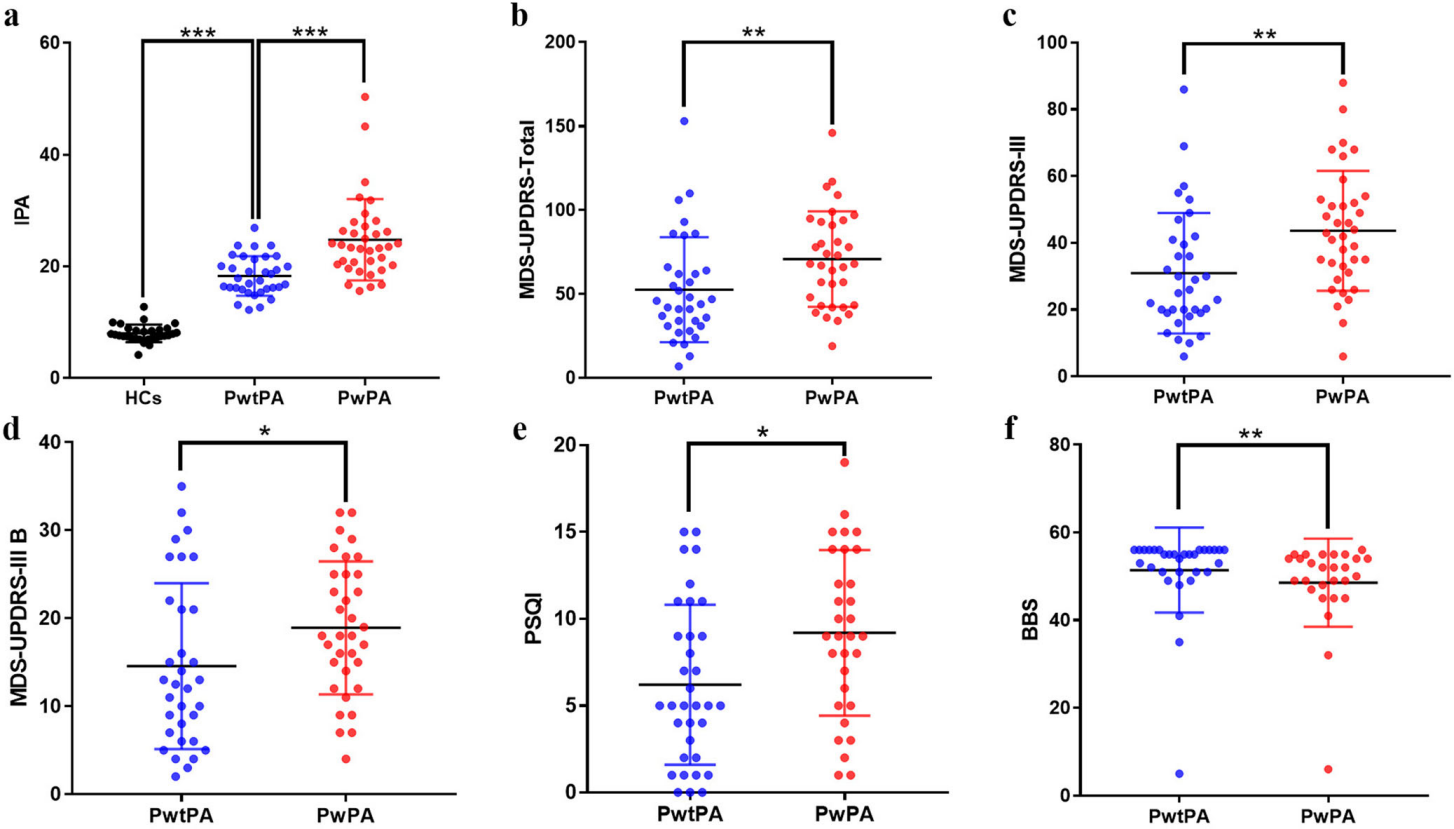
Clinical characteristics of the participants. Comparisons of IPA between PD and HCs (**a**) and comparisons of MDS-UPDRS-Total, MDS-UPDRS-III, MDS-UPDRS-III B, PSQI and BBS characteristics between groups of PwtPA and PwPA (**b**–**f**). Data are mean with error bars representing standard deviation. *p < 0.05, **p < 0.01, ***p < 0.001. *PD* Parkinson’s Disease, *PwtPA* PD without postural abnormalities, *PwPA* PD with postural abnormalities, *HCs* healthy controls, *IPA* the index for postural abnormalities, *MDS-UPDRS* the Movement Disorder Society-Sponsored Revision of the Unified Parkinson’s Disease Rating Scale, *PSQI* the Pittsburgh Sleep Quality Index, *BBS* the Berg Balance Scale.

Data were obtained from 70 PD patients and 30 HCs with a mean age of 68.0 ± 7.3 years and 66.2 ± 7.8 years (*p* > 0.05), respectively. Among the PD with postural abnormalities (PwPA) group, 23 patients had sagittal abnormalities, 9 patients had frontal abnormalities, and 4 patients had both planes of abnormalities. Compared with the PD without postural abnormalities (PwtPA) group, the PwPA group had significantly longer disease duration (5.9 ± 3.6 vs 4.0 ± 3.7 years, *p* = 0.010), significantly higher mean scores on the Hoehn-Yahr scale (2.4 ± 0.7 vs 1.9 ± 0.9, *p* = 0.005), IPA (24.8 ± 7.3 vs 18.3 ± 3.5, *p* < 0.001), MDS-UPDRS-Total (70.8 ± 28.4 vs 52.6 ± 31.2, *p* = 0.005), MDS-UPDRS-III (43.6 ± 18.0 vs 30.9 ± 18.1, *p* = 0.002), MDS-UPDRS-III B (18.9 ± 7.6 vs 14.6 ± 9.4, *p* = 0.041), PSQI (9.2 ± 4.8 vs 6.2 ± 4.6, *p* = 0.013), and lower mean score on BBS (48.6 ± 10.0 vs 51.4 ± 9.7, *p* = 0.006). However, there was no significant difference in gender, age at admission, onset age, body mass index (BMI), first symptom (tremor or rigidity), MDS-UPDRS-III T, MDS-UPDRS-III G, MMSE, NMSS, CSI, HADS, and PDQ-39 between the two groups (*p* > 0.05). Compared with HCs, the PD patients had significantly higher mean value of IPA (21.6 ± 6.6 vs 8.0 ± 1.6, *p* < 0.001) and a higher ratio of male(M)/female(F) (48/22 vs 13/17, *p* = 0.018). The overall magnitude of F1, F2, F3, F4, F5, and F7 were also presented, which were distributed in a step-like manner from low to high among the HC, PwtPA, and PwPA groups (Table 1). The demographic and clinical characteristics of the participants are shown in Table 1 and Fig. 2.

**Table 1:** Demographic and clinical characteristics of the participants.

	All PD (*n* = 70)	PwtPA (*n* = 34)	PwPA (*n* = 36)	HCs (*n* = 30)	*p*
Gender (M/F)	48/22	23/11	25/11	13/17	^a^0.871 ^b^0.018*
Age (years)	68.0 ± 7.3	67.9 ± 7.1	68.2 ± 7.6	66.2 ± 7.8	^a^0.872 ^b^0.264
Onset age (years)	63.1 ± 8.0	63.9 ± 8.1	62.3 ± 8.0	NA	0.408
BMI (kg/m^2^)	23.5 ± 3.2	23.6 ± 3.5	23.4 ± 3.1	23.0 ± 3.2	^a^0.841 ^b^0.486
Disease duration (years)	5.0 ± 3.7	4.0 ± 3.7	5.9 ± 3.6	NA	0.010*
H-Y scale	2.1 ± 0.8	1.9 ± 0.9	2.4 ± 0.7	NA	0.005**
IPA	21.6 ± 6.6	18.3 ± 3.5	24.8 ± 7.3	8.0 ± 1.6	^a^<0.001*** ^b^<0.001***
F1 (°)	7.8 ± 6.6	4.5 ± 4.5	10.9 ± 6.9	1.9 ± 1.7	^a^<0.001*** ^b^<0.001***
F2 (°)	2.0 ± 2.1	1.0 ± 1.0	2.9 ± 2.4	0.3 ± 0.6	^a^<0.001*** ^b^<0.001***
F3 (°)	39.6 ± 13.2	33.8 ± 9.7	45.1 ± 14.0	10.5 ± 4.2	^a^<0.001*** ^b^<0.001***
F4 (°)	24.0 ± 8.3	21.2 ± 4.7	26.8 ± 10.0	7.0 ± 3.6	^a^0.004** ^b^<0.001***
F5 (°)	12.2 ± 8.2	10.2 ± 4.9	14.1 ± 10.2	3.1 ± 2.6	^a^0.048* ^b^<0.001***
F7 (%)	25.7 ± 8.5	22.4 ± 4.3	28.9 ± 10.2	12.5 ± 2.9	^a^0.001** ^b^<0.001***
MDS-UPDRS-Total	61.7 ± 31.0	52.6 ± 31.2	70.8 ± 28.4	NA	0.005**
MDS-UPDRS-III	37.5 ± 19.0	30.9 ± 18.1	43.6 ± 18.0	NA	0.002**
MDS-UPDRS-III B	16.7 ± 8.7	14.6 ± 9.4	18.9 ± 7.6	NA	0.041*
MDS-UPDRS-III T	5.8 ± 5.4	5.5 ± 5.2	6.0 ± 5.6	NA	0.806
MDS-UPDRS-III G	2.6 ± 2.3	2.4 ± 2.7	2.7 ± 1.8	NA	0.119
First symptom (tremor/rigidity)	69 (41/28)	34 (22/12)	35 (19/16)	NA	0.378
MMSE	26.2 ± 5.3	25.8 ± 6.1	26.7 ± 4.3	NA	0.889
NMSS	39.1 ± 25.7	35.3 ± 24.8	43.1 ± 26.5	NA	0.230
PSQI	7.6 ± 4.9	6.2 ± 4.6	9.2 ± 4.8	NA	0.013*
CSI	18.5 ± 14.0	18.8 ± 14.9	18.1 ± 13.8	NA	0.832
HADS	8.4 ± 6.2	8.8 ± 6.1	8.0 ± 6.4	NA	0.613
BBS	50.1 ± 9.9	51.4 ± 9.7	48.6 ± 10.0	NA	0.006**
PDQ-39	26.4 ± 20.4	22.9 ± 18.8	30.0 ± 21.7	NA	0.178

Data are shown as mean ± standard deviation (SD) or count.

*NA* not available, *PD* Parkinson’s Disease, *PwtPA* PD without postural abnormalities, *PwPA* PD with postural abnormalities, *HCs* healthy controls, *BMI* body mass index, *H-Y scale* Hoehn-Yahr scale, *IPA* the index for postural abnormalities, *MDS-UPDRS* the Movement Disorder Society-Sponsored Revision of the Unified Parkinson’s Disease Rating Scale, *MMSE* the Mini-Mental State Examination, *NMSS* the Non-motor Symptoms Scale, *PSQI* the Pittsburgh Sleep Quality Index, *CSI* the Constipation Severity Instrument, *HADS* the Hospital Anxiety and Depression Scale, *BBS* the Berg Balance Scale, *PDQ-39* the 39-item Parkinson’s Disease Questionnaire.

^a^Comparison between the PwtPA group and the PwPA group.

^b^Comparison between the PD patients and HCs.

*p < 0.05, **p < 0.01, ***p < 0.001.

### The correlation between clinical measurements and IPA

**Fig. 3:**
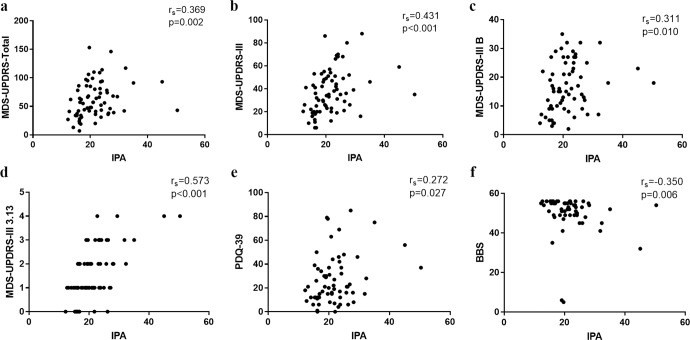
The correlations between clinical measurements and IPA. Spearman’s correlation analysis revealed significant correlations between MDS-UPDRS-Total, MDS-UPDRS-III, MDS-UPDRS-III B, MDS-UPDRS-III 3,13, PDQ-39 and BBS scores and IPA in all PD patients (**a**–**f**). *IPA* the index for postural abnormalities, *MDS-UPDRS* the Movement Disorder Society-Sponsored Revision of the Unified Parkinson’s Disease Rating Scale, *PDQ-39* the 39-item Parkinson’s Disease Questionnaire, *BBS* the Berg Balance Scale.

We examined the correlations between the clinical measurements and IPA via Spearman correlation analysis. As shown in Fig. 3, IPA was positively associated with MDS-UPDRS-Total (*r*_s_ = 0.369, *p* = 0.002), MDS-UPDRS-III (*r*_s_ = 0.431, *p* < 0.001), MDS-UPDRS-III B (*r*_s_ = 0.311, *p* = 0.002), MDS-UPDRS-III 3.13 (*r*_s_ = 0.573, *p* < 0.001), and PSQI score (*r*_s_ = 0.272, *p* = 0.027), but negatively with BBS score (*r*_s_ = −0.350, *p* = 0.006). We further explored and identified significant correlations between IPA and other sub-items of MDS-UPDRS-III (*r*_s_ = 0.241–0.426, *p* = 0.049–<0.001, Table 2).

**Table 2:** Correlation analysis between IPA and other MDS-UPDRS-III sub-items that were significant.

	*r*_s_	*p*
MDS-UPDRS-III 3.3	0.243	0.048*
MDS-UPDRS-III 3.4	0.246	0.044*
MDS-UPDRS-III 3.5	0.250	0.041*
MDS-UPDRS-III 3.6	0.306	0.012*
MDS-UPDRS-III 3.7	0.426	<0.001***
MDS-UPDRS-III 3.8	0.241	0.049*
MDS-UPDRS-III 3.15	0.252	0.041*

*IPA* the index for postural abnormalities, *MDS-UPDRS* the Movement Disorder Society-Sponsored Revision of the Unified Parkinson’s Disease Rating Scale, *r*_s_ Spearman’s correlation coefficient.

*p < 0.05, **p < 0.01, ***p < 0.001.

To determine the proportion of the variance in clinical measurements explained by IPA, simple linear regression models were constructed with clinical measurements significantly correlated to IPA as dependent variables, and IPA as predictor. Linear regression models revealed that IPA contributed significantly to MDS-UPDRS total score (adjusted *R*^2^ = 0.056, *p* = 0.029), MDS-UPDRS-III total score (adjusted *R*^2^ = 0.108, *p* = 0.003), MDS UPDRS-III 3.3 score (adjusted *R*^2^ = 0.046, *p* = 0.045), 3.7 score (adjusted *R*^2^ = 0.099, *p* = 0.006), 3.8 score (adjusted *R*^2^ = 0.055, *p* = 0.031) and 3.13 score (adjusted *R*^2^ = 0.351, *p* < 0.001), and PDQ-39 score (adjusted *R*^2^ = 0.079, *p* = 0.013), respectively (Table 3).

**Table 3:** Linear regression analysis to determine the proportion of the variance in clinical measurements explained by the IPA.

Dependent variables	Adjusted *R*^2^	Unstandardized *β*	Standardized *β*	*p*
MDS-UPDRS-Total	0.056	1.227	0.265	0.029*
MDS-UPDRS-III	0.108	1.001	0.348	0.003**
MDS-UPDRS-III B	0.040	0.308	0.159	0.308
MDS-UPDRS-III 3.3	0.046	0.149	0.246	0.045*
MDS-UPDRS-III 3.4	−0.009	0.035	0.081	0.516
MDS-UPDRS-III 3.5	0.019	0.053	0.184	0.136
MDS-UPDRS-III 3.6	0.030	0.055	0.210	0.088
MDS-UPDRS-III 3.7	0.099	0.088	0.335	0.006**
MDS-UPDRS-III 3.8	0.055	0.069	0.263	0.031*
MDS-UPDRS-III 3.13	0.351	0.096	0.601	<0.001***
MDS-UPDRS-III 3.15	−0.002	0.021	0.113	0.361
PDQ-39	0.079	0.922	0.360	0.013*
BBS	0.004	−0.206	−0.146	0.271

*IPA* the index for postural abnormalities, *MDS-UPDRS* the Movement Disorder Society-Sponsored Revision of the Unified Parkinson’s Disease Rating Scale.

*p < 0.05, **p < 0.01, ***p < 0.001.

### ROC analysis to identify the optimal cut-off value of IPA

**Fig. 4:**
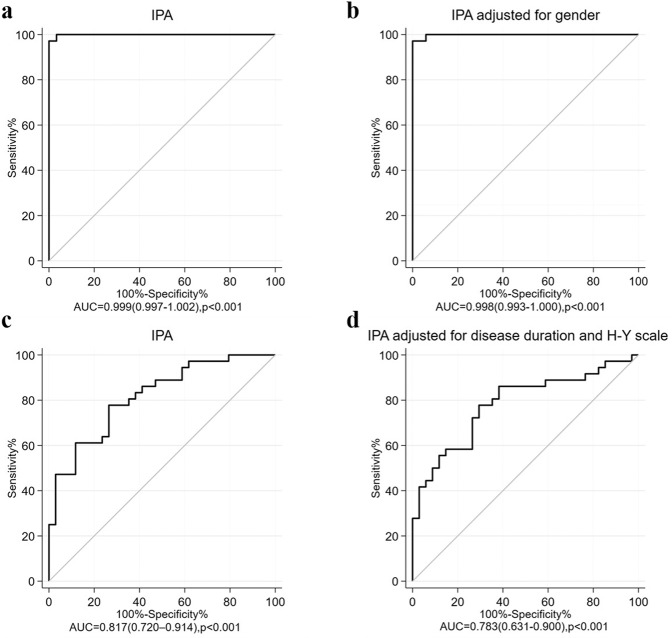
ROC analysis to identify the cut-off value of IPA. The ROC curves for distinguishing PD from HCs adjusted (**b**) and not adjusted for gender (**a**). The ROC curves for distinguishing PwPA from PwtPA adjusted (**d**) and not adjusted for disease duration and H-Y scale (**c**). *PD* Parkinson’s Disease, *PwtPA* PD without postural abnormalities, *PwPA* PD with postural abnormalities, *HCs* healthy controls, *IPA* the index for postural abnormalities, *H-Y scale* Hoehn-Yahr scale.

The ROC curve for the IPA level to distinguish PD from HCs and PwPA from PwtPA is presented in Fig. 4. The optimal cut-off value of the IPA for distinguishing PD from HCs was 12.96, with sensitivity, specificity, AUC, and AUC adjusted for gender of 97.14%, 100.00%, 0.999 (0.997–1.002, *p* < 0.001), and 0.998 (0.993–1.000, *p* < 0.001), respectively (Fig. 4a, b). The optimal cut-off value of IPA for distinguishing PwPA and PwtPA was 20.14 with sensitivity, specificity, AUC, and AUC adjusted for disease duration and Hoehn-Yahr scale of 77.78%, 73.53%, 0.817 (0.720–0.914, *p* < 0.001), and 0.783 (0.631–0.900, *p* < 0.001), respectively (Fig. 4c, d).

## Discussion

In this paper, a summary index, the IPA was introduced for quantifying the postural abnormalities of PD patients and comparing them with HCs. This IPA was obtained based on objective kinematic data derived from Kinect depth camera and it had the merit to summarize the overall quality of an individual’s trunk segment alignment during quiet standing. Notably, the IPA showed important value in distinguishing PD from HCs as well as distinguishing PD with postural abnormalities from those without. In a word, IPA is a useful synthetic index for evaluating postural abnormalities in PD.

Specifically, in this study, the IPA performed surprisingly well in distinguishing PD from HCs with an AUC of 0.999 (0.997–1.002, *p* < 0.001) and adjusted AUC of 0.998 (0.993–1.000, *p* < 0.001). Meanwhile, it provided moderate to high accuracy in distinguishing PwPA from PwtPA with an AUC of 0.817 (0.720–0.914, *p* < 0.001) and adjusted AUC of 0.783 (0.631–0.900, *p* < 0.001). Some researchers have been trying to distinguish PD from healthy individuals or to distinguish PD of different severity stages with the help of kinematic data derived from patients’ gait, posture, and fine movements of the limbs^22,28–30^. Mirelman et al collected multiple gait characteristics obtained with multiple wearable sensors to classify PD motor stages using machine learning methods and they found discriminatory values between motor disease stages with mean sensitivity in the range 72–83%, specificity 69–80%, and AUC 0.76–0.90^31^. A study used normalized stride length (SL) and gait velocity (GV) for recognizing PD using Microsoft Kinect and achieved a high accuracy rate of 97.2%. Though the sample size was relatively small with 18 PD patients and 18 HCs, it suggested the potential use of Microsoft Kinect image and depth sensors for these applications^29^. In this study, we focused on assessment of the global quality of postures and proposed the novel summary index of IPA. Our finding indicated that IPA has important value in distinguishing PD from healthy general population and distinguishing between PD with and without postural abnormalities, which may be helpful in early recognition of PD and early intervention of postural abnormalities for patients.

Previous studies have reported the characteristics of postural abnormalities in PD. For instance, Margraf et al. compared TCC, UCC, and Pisa angles of 192 PD patients and 78 HCs with the free NeuroPostureApp© (http://www.neuroimaging.uni-kiel.de/NeuroPostureApp)^19^. They found that PD patients had a worse posture than HCs in all three angles (*p* < 0.001). For the TCC angle, 39.1% of the patients had a normal posture (<17.4°), 47.9% a presumed stooped posture (>17.4°, <30.2°), and 6.3% had camptocormia (>30.2°). A large comparative study with general population (GPP) revealed that the dropped head angle (DHA), anterior flexion angle (AFA), and lateral flexion angle (LFA) of the thoracolumbar spine were 21.70 ± 14.40°, 13.13 ± 10.79°, and 5.98 ± 12.67° for PD patients and −3.82 ± 4.04°, 0.86 ± 4.25°, and 1.33 ± 2.16° for age-matched GPPs, respectively^4^. Our study disclosed significantly higher values of feature F1, F2, F3, F4, F5, and F7 in PD compared to HCs which was consistent with that study. However, all these features alone only reflect the severity of postural abnormalities of a certain plane. To summarize the global quality of an individual’s body segment alignment during quiet standing, another study introduced the Postural Profile Score Index (PPS)^20^. Twelve joint angles of trunk and of lower limbs, considered representative of the whole-body posture were acquired. The root mean square difference between them and those of the unaffected participants (the Postural Variable Score, PVS) were computed. Then, the PPS was calculated as a combination of the selected PVSs. The authors found significant difference in PPS between PD and HCs (8.59° vs. 6.11°, *p* < 0.001) but did not reveal any correlation of PPS with respect to UPDRS-III or Hoehn-Yahr scale, nor did they propose the cut-off value to distinguish PD from HCs^20^. In this study, the IPA was a combination of the selected features (Fig. 1) and the corresponding feature importance. Therefore, it would be an ideal candidate index for well evaluating and reflecting the overall progress of postural abnormalities in PD from the very beginning.

Moreover, significant weak to moderate correlations were detected between IPA and clinical measurements such as many composite scores of MDS-UPDRS, PDQ-39 score and BBS score (shown in Table 2). Results of linear regression models further confirmed that IPA significantly contributed to MDS-UPDRS total score, MDS-UPDRS-III total score, MDS UPDRS-III 3.3, 3.7, 3.8, and 3.13 score, and PDQ-39 score. Among them, MDS UPDRS-III 3.13 score was mostly explained by the IPA (adjusted *R*^2^ = 0.351, *p* < 0.001), indicating IPA is effective in judging postural abnormalities of PD patients. The MDS-UPDRS together with its composite scores is the most commonly used clinical scale for PD and has been recognized as a reliable and valuable tool to assess the severity of PD^13,32–35^. The PDQ-39 can provide a summary score of the impact of the illness on functioning and well-being and will be useful in the evaluation of the overall effect of different treatments^36–38^. The BBS has been validated to be a valuable screening tool and ongoing assessment tool for patients with PD^39,40^. Results from an observational study of 283 PD patients with ≥5° of forward trunk bending (FTB), lateral trunk bending (LTB), or forward neck bending (FNB) revealed that degree of trunk bending was associated only with motor impairment in LTB (odds ratio [OR], 1.12; 95% confidence interval [CI], 1.03–1.22). ROC curves showed that patients with LTB of 10.5° might have moderate/severe motor impairment^16^. Another two studies demonstrated that camptocormia, antecollis, and Pisa syndrome were associated with severe impairment of neck and back functions, as well as pain in PD patients^41,42^. In our study, weak to moderate correlations were identified between IPA with common PD clinical scales, which indicates that IPA can reflect the clinical severity of postural abnormalities in PD to some extent.

The present study has some limitations. First, the sample enrolled was only composed by a total of 100 participants and it could not be representative of the general population. Second, we did not further verify the results of the study by carrying out prospective researches. In the future, more studies with a much larger sample size and well-controlled homogeneity of participants will be necessary in order to validate this index as a reliable tool in PD patients’ evaluations.

Despite these noted limitations, there are several highlights of this study. First of all, we proposed cut-off values to distinguish PD from HCs and PD with postural abnormalities from those without via global assessment of postures. Moreover, the IPA was obtained based on objective kinematic features derived from Kinect depth camera and computer algorithm which was accurate and repeatable^30,43^. In addition, we adopted various statistical analysis methods to explore correlations between clinical manifestations and IPA which guaranteed the clinical value of IPA.

This study proposed the IPA, a summary index aiding in interpreting the complex and highly interdependent kinematic data, to quantitatively grade the global quality of postural abnormalities in PD. It performed as an effective tool in evaluating the clinical severity of postural abnormalities in PD, as well as distinguishing PD from HCs and PD with postural abnormalities from those without.

## Methods

### Participants

Consecutive patients diagnosed with PD in Tongji Hospital Affiliated to Tongji University from October 2018 to January 2020 were enrolled. The inclusion criteria were: (1) Meeting the 2015 MDS clinical diagnostic criteria for PD^44^; (2) Being able to stand and walk by oneself for 2 min. The exclusion criteria were: (1) Being suspected or diagnosed with Parkinson’s superimposed syndrome or secondary Parkinson’s syndrome; (2) Patients with deformities or injuries that could affect posture; (3) Patients with marked cognitive impairment (Mini-Mental State Examination ≤24). We also recruited age-matched healthy controls (HCs) of 50 to 80 years old from the patients’ relatives. Power Analysis and Sample Size Software (PASS) version 15 (NCSS, LLC, Kaysville, Utah, United States) were used for sample calculation. A sample of at least 23 from the PD group and 12 from the HC group achieves 90% power to detect a difference of 0.300 between the area under the ROC curve (AUC) under the null hypothesis of 0.500 and an AUC under the alternative hypothesis of 0.800 using a one-sided z-test at a significance level of 0.025. We tried to include as many participants as possible and a total 70 PD patients and 30 HCs were enrolled in the end.

All participants gave a written informed consent prior to testing according to the declaration of Helsinki, and the present study was approved by the Ethics Committee of Shanghai Tongji Hospital (Grant Number, 2018-004).

### Device and testing

There are many researchers assessing posture based on Kinect with satisfactory validity and reliability obtained^45–47^. In this study, a Kinect-centered motion analysis device integrating a Kinect v2.0 depth camera (RGB 1920 × 1080 pixels @30fps, depth camera 512 × 424 pixels @30fps, 4-microphone linear phased array, Microsoft) and an independent computer that ran a data capture program was developed by iFLYTEK Suzhou Research Institute^27^. The participants were asked to stand directly in front of the Kinect camera (at a distance of 2 meters)^48^ at ease for 5 s and then actively correct their abnormal posture for 5 s. After that, they were asked to turn left for 90°, relax and stand for 5 s, and then actively correct their abnormal posture for another 5 s. Close-fitting clothing were required and long hair should be tied up^27,49,50^. After the recording, the values of the six selected features F1, F2, F3, F4, F5, and F7 were automatically obtained by computer algorithms. The definition of these features were illustrated in Fig. 1. IPA was calculated with an equation, which was set as follows:

1 $$\begin{array}{l}{\rm{IPA}} = {\rm{F1}} \times 13.2\% + {\rm{F2}} \times 12.6\% + {\rm{F3}} \times 16.5\% + {\rm{F4}} \times 11.3\% \\ \,\qquad\quad+\, {\rm{F5}} \times 6.7\% + {\rm{F7}} \times 40.0\%\end{array}$$

In other words, IPA was defined as the sum of the products of each selected feature and the corresponding feature importance^27^.

Demographic and clinical information such as gender, age at admission, onset age, first symptom (tremor or rigidity) and disease duration were collected. All the PD patients were assessed with the following scales: Hoehn-Yahr scale, the Movement Disorder Society-Sponsored Revision of the Unified Parkinson’s Disease Rating Scale (MDS-UPDRS), the Mini-Mental State Examination (MMSE), the Non-motor Symptoms Scale (NMSS), the Pittsburgh Sleep Quality Index (PSQI), the Constipation Severity Instrument (CSI), the Hospital Anxiety and Depression Scale (HADS), the Berg Balance Scale (BBS), and the 39-item Parkinson’s Disease Questionnaire (PDQ-39). The sub-scores for tremor (items 3.15, 3.16, 3.17, and 3.18), bradykinesia (items 3.4, 3.5, 3.6, 3.7, 3.8, 3.9, and 3.14), and gait (items 3.10, 3.11) were obtained from the MDS-UPDRS-III which were referred to as MDS-UPDRS-III T, MDS-UPDRS-III B, and MDS-UPDRS-III G, respectively. All PD patients performed the assessments 30 min to 2 h after medication intake (based on the participant’s feedback when they usually experience best ON) in ON medication condition^51^. The PD patients were further divided into group with postural abnormality (PwPA) and group without postural abnormality (PwtPA) based on whether the MDS-UPDRS-III 3.13 item was greater than or equal to 2 points^10^.

### Statistical analysis

Quantitative data were shown as mean ± standard deviation (SD). The normality of distribution of demographic and clinical data was initially tested using the Kolmogorov–Smirnov test. The student’s *t* test and the Mann–Whitney U test were used for comparison of normally and abnormally distributed data, respectively. The Chi-square test was used to evaluate the differences in categorical variables. Spearman’s correlation analysis was conducted to explore correlations between IPA and other clinical data. A correlation coefficient of 0.00–0.10 indicates negligible correlation, 0.10–0.39 indicates weak correlation, 0.40–0.69 indicates moderate correlation, 0.70–0.89 indicates strong correlation, and 0.90–1.00 indicates very strong correlation^52^. Linear regression was used to determine the proportion of the variance in clinical measurements explained by the IPA. Receiver operating characteristic (ROC) curves were operated to estimate the optimal IPA cut-off values which were determined by maximizing the Youden’s index on the ROC curve. An area under the curve (AUC) value <0.7 indicates a low diagnostic accuracy, 0.7–0.9 indicates moderate accuracy, and >0.9 indicates high accuracy^53^. Statistical analyses were performed using GraphPad Prism version 7 (Graph Pad Software Inc, San Diego, CA, USA) and Stata version 16 (StataCorp, College Station, TX, USA), and significance was set at a two-tailed *p* value < 0.05.

## Data Availability

The data that support the findings of this study and the algorithms to extract the features mentioned in the article are available from the corresponding author upon reasonable request.
